# Clinical and imaging characteristics of PFO‐related stroke with different amounts of right‐to‐left shunt

**DOI:** 10.1002/brb3.1122

**Published:** 2018-10-11

**Authors:** Dan He, Qian Li, Guangjin Xu, Zheng Hu, Xuefei Li, Yinping Guo, Shabei Xu, Wei Wang, Xiang Luo

**Affiliations:** ^1^ Department of Neurology, National Key Clinical Department and Key Discipline of Neurology, The First Affiliated Hospital Sun Yat‐sen University Guangzhou Guangdong China; ^2^ Department of Neurology, Tongji Hospital, Tongji Medical College Huazhong University of Science and Technology Wuhan Hubei China; ^3^ Department of Neurology Shenzhen Shekou People’s Hospital Shenzhen China; ^4^ Department of Obstetrics and Gynecology, The First Affiliated Hospital Sun Yat‐sen University Guangzhou Guangdong China

**Keywords:** cryptogenic stroke, infarct pattern, patent foramen ovale, right‐to‐left shunt

## Abstract

**Introduction:**

Right‐to‐left shunt (RLS) induced by a patent foramen ovale (PFO) is associated with an increased risk of cryptogenic stroke (CS). However, little is known about the relationship between the amount of RLS and the stroke pattern. In this study, we aimed to evaluate the distinct clinical features of PFO‐related CS in different RLS degrees resulting from PFO.

**Methods:**

This is a cohort study of 222 CS patients admitted to the Tongji Hospital from 1st May 2014 to 31st April 2017. All patients underwent contrast transcranial Doppler examination. And then, 121 (54.5%) were classified as non‐RLS group, while 76 (34.2%) were classified as mild RLS group and 25 (11.3%) were large RLS group according to the number of micro‐emboli signals. The groups were compared with respect to their clinical and neuroimaging characteristics.

**Results:**

In terms of risk factors of stroke, the prevalence of hypertension was lower in mild group (*p* = 0.002). Regarding the infarct patterns in different CS patients, we found that the multiple cortical lesions were more frequently observed (*p*<0.001) with increasing RLS in DWI. Moreover, there was a rising trend in the proportion of small lesions (≤1 cm) with an increasing RLS (*p* < 0.01). And as RLS increased, the posterior circulation was more likely to be influenced (*p* < 0.05). In addition, the serum cholesterol concentration was lower in the large RLS group, compared to the non‐RLS group (*p* = 0.003) and mild RLS group (*p* = 0.008). While the mean platelet volume (MPV) in mild group was significantly higher than that of non‐RLS group (*p* = 0.013).

**Conclusion:**

Patients with larger RLS show more infarction in posterior circulation, higher frequency of small lesions or multiple cortical lesions. The results of our study indicate that the infarct patterns might be a clue of PFO‐related stroke.

## 1 INTRODUCTION

Approximately 50% (ranged from 40% to 56%) of cryptogenic stroke (CS) can be found having patent foramen ovale (PFO) (Hara et al., 2005; Lechat et al., 1988; Webster et al., 1988). PFO is established as an important pathogenesis of CS (Fisher & McAllister, 2015; Overell, Bone, & Lees, 2000; Yaghi, Bernstein, Passman, Okin, & Furie, 2017), and three recent randomized clinical trials demonstrated that percutaneous closure of PFO was better than medical therapy alone for the prevention of recurrent ischemic stroke (Mas et al., 2017; Saver et al., 2017; Sondergaard et al., 2017).

Previous studies have implied that PFO with specific characteristics was associated with the onset of stroke, including the size or height of PFO, degree of right‐to‐left shunt (RLS), present of concomitant atrial septum aneurysm, and so on (Akhondi et al., 2010; Lee et al., 2010; Mas et al., 2001). Thus, knowledge of these factors could assist clinicians in identifying populations susceptible to initial or recurrent stroke due to PFO and evaluating the necessity of aggressive treatments.

Contrast TCD is an alternative method for PFO diagnosis. Based on intracranial detection of intravenously injected micro‐emboli, the test is performed with normal respiration and the Valsalva maneuver to maximize sensitivity and specificity of the results (Silvestry et al., 2015). This inspection technique is easier for quantifying and assessing the size as well as functional relevance of RLS (Droste et al., 2000; Sloan et al., 2004). Besides, due to its noninvasive nature, the method is highly tolerable and acceptable for the patients (Komar et al., 2014).

The aim of this study was to analyze the association between the amount of RLS induced by PFO and the onset of CS by comparing clinical characteristics, neuroimaging data, and laboratory test results in different RLS groups.

## 2 MATERIAL AND METHODS

### 2.1 Study population

Patients attending the stroke unit at Wuhan Tongji Hospital diagnosed with acute ischemic stroke were consecutively recruited in this study from 1st May 2014 to 31st April 2017. This study was approved by the ethics board of Tongji Hospital, and all participations or their relatives provided informed consent.

Collection of basic information on gender, age, onset time, symptoms, physical signs, personal history (smoking, alcohol intake, etc.), and previous medical history (including migraine, hypertension, hyperlipidemia, diabetes mellitus, prior stroke/transient ischemic attack, TIA) was performed in all enrolled patients. All patients underwent standard laboratory tests for etiological diagnosis, which included routine blood tests, blood glucose, glycosylated hemoglobin, homocysteine, coagulation function, electrocardiogram/holter, chest radiograghy/computed tomography (CT), vascular ultrasound Doppler of lower limbs, transthoracic echocardiography (TTE), contrast transcranial Doppler (c‐TCD), brain imaging (brain nuclear magnetic resonance imaging (MRI) including T1 sequences, T2 sequences, diffusion‐weighted imaging (DWI) sequences, and fluid‐attenuated inversion recovery (FLAIR) sequences), and intracranial vascular angiography (digital subtraction angiography, DSA; computed tomography angiography, CTA; or magnetic resonance angiography, MRA).

In our study, all patients were classified as having one of the following cause of stroke according to the Trial of Org 10172 in Acute Stroke Treatment (TOAST) criteria, including large artery atherosclerosis (LAA), cardioembolism (CE), small vessel occlusion (SVO), stroke of other determined etiology (SOE), or stroke of undetermined etiology (SUE) (Adams et al., 1993). CS was limited to the case in which two or more causes identified or without no clear cause (Adams et al., 1993).

We chose c‐TCD as the method for RLS detection (Komar et al., 2014).

### 2.2 c‐TCD protocol and RLS subgroup

As described previously, contrast agent was made with 9 ml saline, 1 ml air, and one drop of the patient's blood, which were adequately mixed between two 10‐ml syringes connected by a three‐way stopcock. The drop of blood was used to extend the suspension time of air micro‐emboli in the blood to improve the sensitivity of detecting the micro‐emboli signal (MES). The solution was injected rapidly into the antecubital vein during normal respiration and 5 s prior to the start of Valsalva maneuver; the RLS was quantified by counting the number of MES in the middle cerebral artery observed in the first 25 s after injecting the solution. For better analysis, we reallocated these patients into three groups: non‐RLS: no MES, mild RLS: ≤25 MES, and large RLS: >25 MES. Then, to subsequently differentiate the RLS caused by intracardiac and intrapulmonary shunting detected by c‐TCD, transesophageal echocardiography (TEE) was performed when more than 10 MES were detected (Serena et al., 1998; Telman et al., 2008). TTE were also carried out for all patients to excluded other diseases that can induce RLS, such as atrial septal aneurysm, persistent Eustachian valve, chiari network, atrial septum defects, and cyanotic congenital heart defects (Aypar, Sert, & Odabas, 2013; De Vecchis, Baldi, Ariano, Giasi, & Cioppa, 2017; Schuchlenz, Saurer, Weihs, & Rehak, 2004), and furthermore, patients may undergo pulmonary CTA if intrapulmonary shunt diseases such as pulmonary arteriovenous malformation (Kucukoglu et al., 2012) were suspected.

### 2.3 Neuroimaging Assessment

All patients underwent brain MRI within 7 days of the stroke onset. Data were obtained from the T1‐ and T2‐weighted sequences, DWI sequences, and FLAIR sequences. The inspection images were reviewed by experienced neuro‐radiologists who were blinded to the study groups and the existence of PFO.

**Figure 1:**
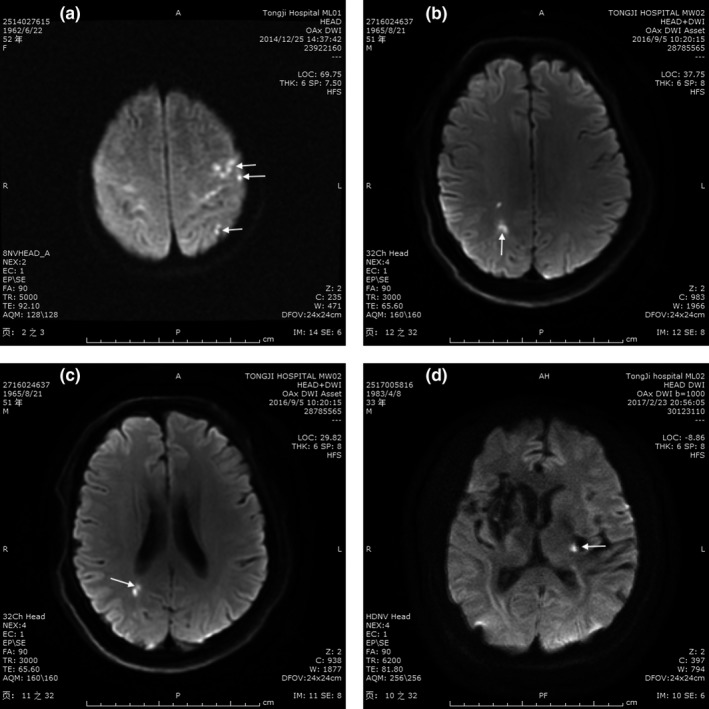
Representative cases showing different infarct patterns in diffusion‐weighted imaging (DWI). (a) Multiple small lesions located in cortex; (b) multiple small corti‐subcortical lesions; (c) multiple small subcortical lesions; (d) single small deep lesion (white arrows)

The ischemic lesions were first classified as single lesion and multiple lesions by the lesion numbers. In terms of location, lesions were divided into cortical, subcortical, cortical–subcortical, and deep white matter lesions (Table 1) (Thaler et al., 2013). Further, the stroke lesions were classified as small lesions (infarction diameter ≤1 cm), large lesions (infarction diameter >1 cm), and large hemisphere infarctions (infarction diameter >3 cm, or involving at least 2 anatomic site of main blood vessel territories) according to the size of lesions (Kim, Kim, et al., 2013; Zha, Sari, & Torbey, 2015). According to the vascular territory involved, infarctions were divided into anterior circulation, posterior circulation, and both anterior and posterior circulation and bilateral anterior circulation (Kang, Chalela, Ezzeddine, & Warach, 2003). Some specific samples of stroke pattern are shown in Figure 1.

**Table 1:** Classification of infarct lesions according to the location

Cortical lesion	Gray matter of frontal lobe, parietal lobe, temporal lobe, limbic lobe, or cerebellar hemispheres
Subcortical lesion	White matter of frontal lobe, parietal lobe, temporal lobe, limbic lobe, or cerebellar hemispheres
Cortical–subcortical	Across both gray matter and white matter
Deep white matter	Internal capsule, Corona radiate, Centrum semiovale, Caudate nucleus, Globus pallidus, Putamen, mesencephalon, Thalamus, pons, or cerebellar vermis

### 2.4 Statistical Analysis

The data were analyzed using SPSS version 23 for Mac (SPSS Inc., Chicago, IL, USA). Continuous variables with normal distribution were presented as mean ± *SD*, whereas nominal variables were shown as numbers (percentages). For the continuous variables, one‐way ANOVA was used to compare differences among groups and L‐S‐D test was chosen as the method of post hoc multiple comparisons for intergroup differences. For nominal variables, the chi‐square test was used to analyze intergroup differences, and chi‐square test for trend was used to determine whether the proportion of patients with different infarct patterns changed significantly over the degree of RLS. A *p*‐value <0.05 was considered statistically significant. Bonferroni method was used to correct type I errors in multiple comparisons.

## 3 RESULTS

### 3.1 Clinical characteristics

Among the 673 patients who were admitted with acute ischemic stroke, 222 patients (32.99%) met the inclusion criteria. Of these, 121 patients had non‐RLS, 76 mild RLS, and 25 large RLS. The mean age was 52.5 ± 13.2 in patients without RLS, 53.9 ± 13.4 with mild RLS, and 54.0 ± 13.2 with large RLS; there was no significant difference of the mean age among groups. In terms of risk factors of stroke, we observed significant differences in the prevalence of hypertension (*p* = 0.004) and proportion of cigarette smokers (*p* = 0.032) among the three groups. When compared to the non‐RLS group, the prevalence of hypertension was significantly lower in the mild group (*p* = 0.002), while the proportion of the cigarette smokers was distinctly higher (*p* = 0.009). No obvious difference was found in the average onset age, gender, prevalence of diabetes mellitus, prior stroke/TIA, and alcohol intake history (Table 2).

**Table 2:** Clinical characteristics of CS patients with different degrees of RLS

	Non‐RLS (no MES)	Mild RLS (≤25MES)	Large RLS (>25 MES)	*p*‐Value^a^
Age (mean±SD), y	52.5 ± 13.2	53.9 ± 13.4	54.0 ± 13.2	0.72
Gender, male, *n* (%)	89 (73.6)	64 (84.2)	20 (80.0)	0.207
Vascular risk factors				
Hypertension, *n* (%)	65 (53.7)	23 (30.3)^b^	9 (36)	0.004
Diabetes, *n* (%)	20 (16.5)	18 (23.7)	7 (28)	0.284
Hyperlipidemia, *n* (%)	56 (46.3)	34 (44.7)	15 (60)	0.393
Smoking, *n* (%)	57 (47.1)	50 (65.8)^b^	12 (48)	0.032
Alcohol intake, *n* (%)	44 (36.4)	30 (39.5)	10 (40)	0.883
Migraine, *n* (%)	10 (8.3)	5 (6.6)	4 (16)	0.339
Prior stroke/TIA, *n* (%)	13 (10.8)	13 (17.1)	4 (16)	0.414
Recurrent stroke, *n* (%)	12 (9.9)	9 (11.8)	3 (12)	0.896
Onset age <55 y, *n* (%)	68 (56.2)	40 (52.6)	10 (40)	0.334

Notes

TIA: transient ischemic attack.

a *p* values by one‐way ANOVA for homogeneity or Pearson chi‐square test to compared proportions across different RLS groups.

b *p* < 0.01, for intergroup comparisons between non‐ and mild RLS groups by Pearson chi‐square test.

### 3.2 Infarct pattern

**Figure 2:**
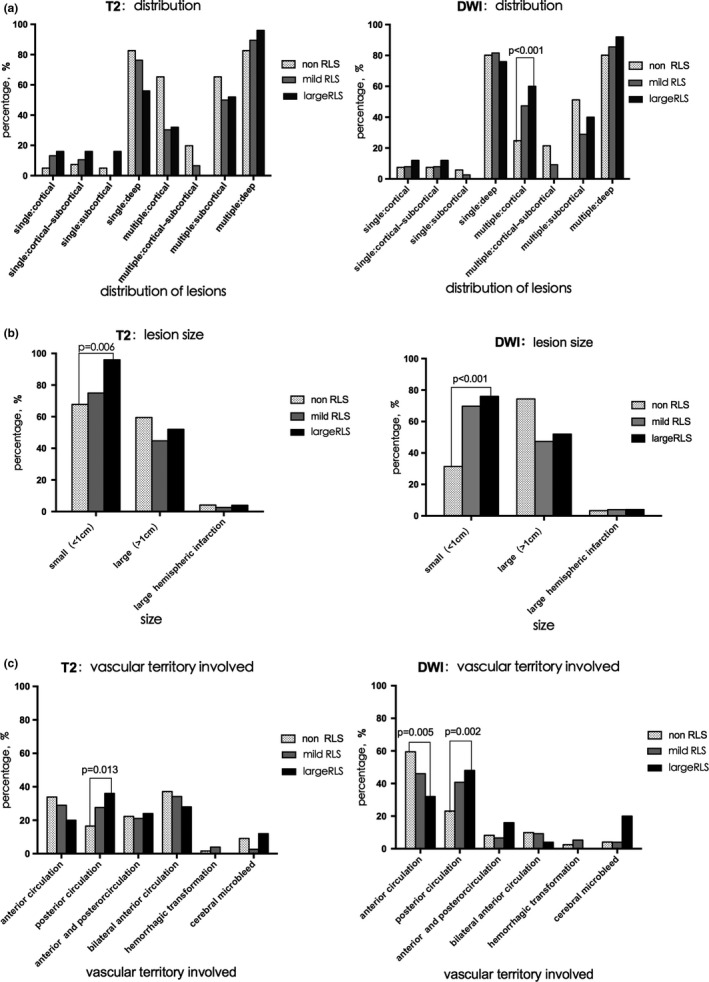
Comparisons of infarct patterns among CS patients in different RLS groups in T2 and DWI sequences. (a) Lesion distributions of patients in different RLS groups; (b) lesion size of patients in different RLS groups; (c) vascular territory involvement of patients in different RLS groups. *p* values were presented for chi‐square test for trend

We compared and analyzed the infarct patterns of CS patients with different RLS in both DWI and T2 sequences. For DWI lesions, the distribution of lesions was different depending on the amount of RLS. As shown in Figure 2a, the proportion of patients with multiple cortical lesions was 30/121 (24.79%) in non‐RLS group, 36/76 (47.37%) in mild RLS group, and 15/25 (60.00%) in large RLS group. Chi‐square analysis for trend shows an increasing trend with increasing RLS (chi‐square for trend = 16.490; *p* < 0.001). With respect to the lesion size (Figure 2b), the proportion of small lesions (≤1 cm) varied from 31.40% (38 out of 121 patients) in non‐RLS group, 69.74% (53 out of 76 patients) in mild RLS group, and 76.00% (19 out of 25 patients) in large RLS group with a statistically increasing trend from non‐RLS group through mild RLS group to large RLS group (chi‐square for trend = 31.104; *p* < 0.001). Besides, when the proportions of patients with lesions in different vascular territories were compared among different RLS groups (Figure 2c), there were a trend for increased percentages of posterior circulation involved (23.14% in the non‐RLS group, compared to 40.79% in the mild RLS group and 48.00% in the large RLS group, chi‐square for trend = 9.468; *p* = 0.002), and a trend for reduced involvement of anterior circulation (59.50% in the non‐RLS group, compared to 46.05% in the mild RLS group and 32.00% in the large RLS group, chi‐square for trend = 7.771; *p* = 0.005), with the increasing amounts of RLS. Finally, no difference in the lesion number was noted among groups.

We also analyzed the T2 lesions. The number and distribution of lesions did not differ depending on the amount of RLS. Similar to the results of characteristics in DWI, the proportion of patients with small lesions (Figure 2b) increased significantly (chi‐square for trend = 7.612; *p* = 0.006) from 67.77% (82 out of 121 patients) in the non‐RLS group to 75.00% (57 out of 76 patients) in the mild RLS group and 96.00% (24 out of 25 patients) in the large RLS group. While a linear trend of increase in the proportion of patients with posterior circulation (Figure 2c) involvement was also observed (16.53% in non‐RLS group to 27.63% in the mild group and 36.00% in the large group, *p* = 0.013 for trend).

### 3.3 Laboratory tests

Finally, we analyzed the characteristics of laboratory tests and observed that the level of cholesterol was remarkably lower in the large RLS group, compared to the non‐RLS group and mild RLS group. We also found mean platelet volume (MPV) and international normalized ratio (INR) differed significantly among the three groups, and the MPV in mild RLS group was significantly higher than that of the non‐RLS group (11.46 ± 3.50 vs. 10.57 ± 1.57, *p* = 0.013). However, we did not find any obvious trend in these indexes over the amount of RLS. Data are presented in Table 3.

**Table 3:** Laboratory tests in CS patients with different degrees of RLS

	Non‐RLS (no MEBs)	Mild RLS (≤25 MEBs)	Large RLS (>25 MEBs)	*p* a
Cholesterol, mmol/l	3.91 ± 0.96	3.86 ± 1.07	3.25 ± 0.75^b^^,^ c	0.010
MPV, fl	10.57 ± 1.54	11.46 ± 3.50^d^	10.86 ± 1.62	0.046
INR	1.04 ± 0.11	1.07 ± 0.16	1.14 ± 0.26^e^	0.023

INR: international normalized ratio; MPV: mean platelet volume.

a *p* values by one‐way ANOVA to compared proportions across different RLS groups.

b *p* = 0.003, when compared with non‐RLS group by post hoc multiple comparisons of L‐S‐D test.

c *p* = 0.008, when compared with mild RLS group by post hoc multiple comparisons of L‐S‐D test.

d *p* = 0.013, when compared with non‐RLS group by post hoc multiple comparisons of L‐S‐D test.

e *p* = 0.008, when compared with non‐RLS group by post hoc multiple comparisons of L‐S‐D test.

Besides, no statistical significant difference was found in other indexes such as levels of triglycerides (TG), high‐density lipoprotein, low‐density lipoprotein, homocysteine, blood sugar, glycosylated hemoglobin, blood platelet counts, platelet distribution, coagulation function, and hemorheology among the three groups.

## 4 DISCUSSION

Based on our knowledge, there is a connection between the infarct pattern in MRI and stroke etiology as a stroke caused by different pathogenies may have their own specific infarct pattern (Santamarina et al., 2006). Several former studies have documented the relationship of PFO structural or functional factors and ischemic stroke neuroimaging characteristics (Akhondi et al., 2010; Bonati et al., 2006; Kim, Sohn, et al., 2013; Santamarina et al., 2006), but results of these studies were controversial and contradictory. In the present study, we focused on the infarct pattern of small lesion prevalence, vascular territory involved, and distribution of the infarct lesions. We demonstrated that the degree of RLS induced by PFO was associated with the stroke patterns in DWI and T2 sequence, and the trend was more obvious in DWI sequence. Specifically, we measured the acute infarct lesions by DWI sequence and the cumulative infarct lesions by combining the T2 sequence. Our results showed that a higher amount of RLS was correlated with a higher percentage of small lesions and a more likely involvement of the posterior circulation. Furthermore, the frequency of multiple cortical lesions in DWI sequence was higher when the amount of RLS increased.

The results can be explained as follows. First, although the pathogenesis of PFO‐induced stroke is unclear, paradoxical thromboembolism (PTE) is one of the widely accepted hypothesis (Elmariah et al., 2014; Homma, Sacco, Di Tullio, Sciacca, & Mohr, 2002; Jung et al., 2013; Overell et al., 2000). PFO is considered as a channel for the embolus to travel from the venous system to cerebral circulation, which can generally only allow the smaller ones to pass through (Kim, Kim, et al., 2013). Therefore, if the RLS increases, the number of small emboli traveling through the PFO can relatively increase, and this can provide an anatomic basis of multiple intracranial small lesions. A former study indicated that the size of PFO measured by TEE was positively correlated with the amount of RLS detected by c‐TCD; the larger the PFO is, the higher is the number of microbubbles (Telman et al., 2008). Hausmann D et al. have shown that a larger size of PFO could allow a few larger emboli to pass through, which may be more likely to cause larger lesions (Hausmann, Mugge, & Daniel, 1995). However, a few other studies have demonstrated that large lesions of PFO‐related CS were more likely to correlate with the existence of atrial septum aneurysm (Bonati et al., 2006), septal excursion distance (Akhondi et al., 2010), venous thrombosis, and coagulation disorders (Pezzini et al., 2009; Tohgi, Kawashima, Tamura, & Suzuki, 1990), but have no obvious connection to the size of PFO, or the degree of RLS (Akhondi et al., 2010; Bonati et al., 2006). Second, it has been proved in the previous study that blood flow of posterior circulation significantly exceeded that of anterior circulation in patients with PFO when taking Valsalva maneuver (Hayashida et al., 2001). This finding explains our result that it is more likely to influence the posterior circulation when the amount of RLS induced by PFO increases.

Furthermore, with advances in the sensitivity and accuracy of inspection equipment, a higher prevalence of subclinical atrial fibrillation (AF) was detected (Flint, Banki, Ren, Rao, & Go, 2012; Ritter et al., 2013). Previous studies have demonstrated that the lesions of AF‐relate ischemic stroke were more frequently observed in the cortical–subcortical territory (Kim, Sohn, et al., 2013), while in our study, the PFO‐related CS was more often observed as multiple cortical lesions and had a positive correlation with the amount of RLS. This indicates that the mechanism of PFO‐related CS varies from that of cardiogenic stroke.

Currently, the pathogenic mechanism of PFO‐related CS is still controversial, and PTE is one of the most commonly accepted mechanisms (Windecker, Stortecky, & Meier, 2014). The incidence of PTE is strongly linked to RLS (Kent et al., 2013; Overell et al., 2000; Wessler et al., 2015). If the degree of RLS improves, the possibility of ischemic stroke is correspondingly higher. As a result, combining the recent research, we deduce the pathogeny of PFO‐related CS with a larger amount of RLS is more closely associated with PTE. In other words, the infarct pattern of PTE induced by PFO is more often observed as multiple small cortical lesions, and lesions involved the posterior circulation. On the other hand, in CS patients with lower amounts of RLS, there may be other mechanisms apart from PTE, which need further investigation.

Another strength of our study is the usage of T2 sequence to explore the infarct patterns of PFO‐related CS. Despite the DWI sequence has been widely used to explore the infarct patterns of PFO‐related CS (Bonati et al., 2006; Boutet et al., 2014; Jung et al., 2013; Kang et al., 2003; Kim, Sohn, et al., 2013), some PFO‐related CS patients might be neurologically asymptomatic by this sequence (DWI could not detect the old or asymptomatic lesions). Moreover, we simultaneously collected data from FLAIR sequence for distinguishing small infarcts from Virchow–Robin spaces and other misleading signals (Liu et al., 2009). From our study, the significant trends in the prevalence of small lesion and involvement of posterior circulation in T2 sequences were consistent with those in DWI sequences, while no trend was observed in the percentage of multiple cortical lesions.

The reason of the discrepancy implies that stroke onset is originated from complicated responsible mechanisms (Adams et al., 1993); as presented above, ischemic stroke patients with two or more causes were enrolled in our study. It was difficult to determine the definite etiology in these patients. And moreover, additional mechanisms apart from PTE, which remain undescribed and poorly understood, could participate in the pathogenic process of PFO‐related stroke (Windecker et al., 2014).

In this study, we also investigated the relationship of RLS severity with the demographic and serological characteristics of CS patients. Our results showed a lower prevalence of hypertension in CS patients with mild RLS and lower cholesterol level in patients with large RLS. Hypertension and dyslipidemia were acknowledged playing pivotal role in atherosclerosis and cardiovascular risk (Hurtubise et al., 2016; Weber & Noels, 2011), and thus, it implied from our study that atherosclerosis played little role in PFO‐related stroke. Then, we analyzed the laboratory test data in our study. In line with an earlier study which demonstrated that the MPV decreased after percutaneous closure of PFO (Duzel et al., 2014), we found the MPV index was significantly higher in the mild RLS group than that in non‐RLS group. Based on this, we speculated that platelet activity might be involved in the pathogenesis of stroke induced by mild RLS and need more thorough investigations. Moreover, we also observed that patients with large RLS had a higher INR value than patients without RLS； the reason was still unclear now. INR is an index reflecting the coagulation function. A higher value stands for longer blood coagulation time, indicating a higher hemorrhagic tendency (Smith et al., 2006). In the present study, although there were no statistical trends in the percentages of hemorrhagic transformation or microbleeding over the increasing of RLS, we did observe more hemorrhagic lesions in the large RLS group when compared to the other two groups, so we suggested that PFO‐related CS patients with large RLS may have a greater risk of hemorrhage. This finding could provide the basis of personalizing the best clinical treatment and assessing the prognosis risk in CS patients with large RLS induced by PFO.

There were a few limitations in our study. First, this study was retrospective and carried out in a single hospital. Thus, further in‐depth prospective and multicenter studies are needed to illustrate the stroke mechanisms considering the different degree of RLS induced by PFO. Second, we enrolled a consecutive series of unselected patients with an average age of 53.2 ± 13.2 (range from 7 to 83). That made it difficult to distinguish the PFO‐related lesions and those originated from other reason, especially atherosclerosis, in patients of an older age. That may be why the infarct pattern on T2 sequence was not totally consistent with that on DWI sequence. However, the previous study indicated that the presence of PFO was independently associated with stroke in both younger and older ages (Handke, Harloff, Olschewski, Hetzel, & Geibel, 2007), and the diameter of PFO would increase with age (Hagen, Scholz, & Edwards, 1984). In addition, the prevalence of venous thrombosis and pulmonary embolism (Stollberger et al., 1993), as well as the presence of concomitant atrial septum aneurysm (Handke, Harloff, Bode, & Geibel, 2009), which may act as important risk factors of PTE when coexistence with PFO (Mas et al., 2001; Stollberger et al., 1993), would also increase with age. And besides, previous studies demonstrated some hemodynamic changes elevating RLS in elderly patients, the promoting pulmonary–arterial pressure for instance, could contribute to a higher possibility of PTE (Homma, DiTullio, Sacco, Sciacca, & Mohr, 2004; Ueda et al., 2004). We speculated that the importance of PFO in the pathogenesis of stroke was more obvious in aged patients.

In summary, we found an association between the infarct pattern along with the degree of RLS. We showed that small, posterior circulation and multiple cortical lesions could be more likely resulted from PFO. In addition, our results also indicate that the stroke mechanism is different depending on the degree of RLS caused by PFO.

## CONFLICT OF INTEREST

The authors have declared that no conflict of interest exists.

## AUTHOR CONTRIBUTIONS

D.H., Q.L., W.W., and X.L. were responsible for designing the experiments; H.Z., X.L, Q.L, G.J.X, Y.G., S.X., and D.H. were responsible for acquiring and analyzing the data; D.H., Q.L., W.W., and X.L. were responsible for drafting the manuscript figures. All the authors have reviewed, revised, and approved the final manuscript.
